# Synthesis of Valid Camera Poses for the Inspection of Triangular Facets in a 3D Mesh

**DOI:** 10.3390/s23187878

**Published:** 2023-09-14

**Authors:** Edward Parrott, Joshua K. Pickard, Rickey Dubay

**Affiliations:** 1 Department of Mechanical Engineering, University of New Brunswick, Fredericton, NB E3B 5A3, Canada; dubayr@unb.ca; 2 Eigen Innovations Inc., Fredericton, NB E3B 1S1, Canada; josh.pickard@eigen.io

**Keywords:** quality inspection, interval analysis, camera models, intelligent inspection, constraint satisfaction, advanced manufacturing

## Abstract

Automation of visual quality inspection tasks in manufacturing with machine vision is beginning to be the de facto standard for quality inspection as manufacturers realize that machines produce more reliable, consistent and repeatable analyses much quicker than a human operator ever could. These methods generally rely on the installation of cameras to inspect and capture images of parts; however, there is yet to be a method proposed for the deployment of cameras which can rigorously quantify and certify the performance of the system when inspecting a given part. Furthermore, current methods in the field yield unrealizable exact solutions, making them impractical or impossible to actually install in a factory setting. This work proposes a set-based method of synthesizing continuous pose intervals for the deployment of cameras that certifiably satisfy constraint-based performance criteria within the continuous interval.

## 1. Introduction

While the implementation of quality inspection procedures on manufacturing lines is a well-established practice with a long history, the use of automated methods and machine vision techniques is a relatively new area of study. Central to this new area is the effective deployment of cameras for image capture; however, this is often left to the experience of engineers or technicians to pick what they feel is the best deployment for the task. In most cases, the hardware is purchased beforehand, based on some initial assumptions that later prove to be incorrect. While good deployment results are often possible with this approach, they lack mathematical rigour and quantifiable parameters that could be used to analyze the deployment and objectively assess its effectiveness at inspecting the desired object.

**Figure 1:**
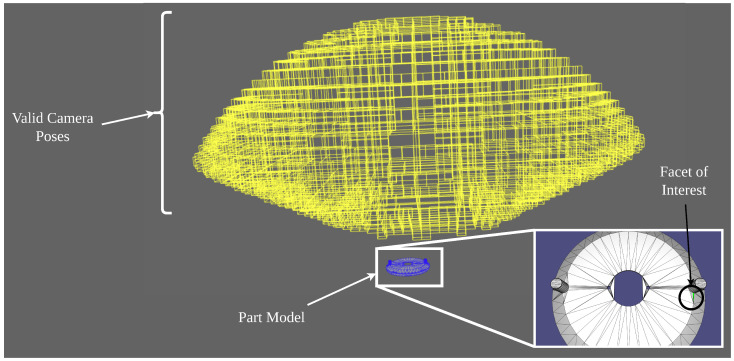
Part mesh representation and valid camera deployment poses for a given facet.

Methods have been devised for the synthesis of optimal deployments; however, these are often extremely limited in their scope and are thus theoretical exercises with no ability to be practically implemented in a real factory setting. This research aims to address this gap in the literature by proposing a set-based method of synthesizing continuous pose intervals for camera deployments, which can be certified to completely inspect any section of a part’s surface for any pose contained within the prescribed interval. Representative results can be seen in Figure 1. This not only allows camera deployment solutions to be quantified, but also allows for their actual implementation, as they allow for realistic integration errors during installation.

The problem of computing complete and realistic inspection spaces for large areas of a part’s surface is complex and multi-faceted, and, as such, the research contained herein imposes the following conditions to limit the scope of this preliminary exploration: the part will be represented as a triangular mesh, the valid inspection poses will be solved for a single triangle from this mesh and idealized models will be used for the inspection camera.

## 2. Problem Definition and Terminology

It is first useful to establish some definitions of terms that appear frequently in the literature and use these to frame the problem.

The first, and most important, term is the inspection task. The inspection task is defined in [1] as a “measurement, or set thereof, to be performed by a given sensor on some features of an object, for which a geometric model is known”. Likewise, in this work, the inspection task is defined as the surfaces of the part that need to be acceptably imaged to perform a suitable inspection. The method for planning the inspection task will be, as stated in [1], based on a known geometric model of the object and a set of inspection constraints that define valid inspection regions for the given inspection camera.

Next, it is important to define what is meant by the model. The model is the 3D data representation of the part’s geometric structure. While early attempts to solve this problem made use of parametric expressions of part geometry [1,2], it has since become the standard in the literature to use tessellated model formats [2,3,4,5]. This means that instead of representing a part as a set of parameterized surfaces and edges, it is represented by a mesh, or tessellation, of small polygons. The format most commonly used in these formulations is a triangular mesh. A triangular mesh is a representation of a given part geometry as a triangular mesh with edges, faces, and vertices defining the 3D structure of the part surfaces [6]. As a visual representation of this concept, Figure 1 shows a part’s triangular mesh representation. Each vertex in the mesh is described as a set of 3D coordinates, and these coordinates are compiled in a list of vertices. Each triangular facet is then described as a set of three vertices (edges are described by a set of two vertices). Additionally, each vertex and face has an outward-facing normal vector assigned to it [2,3,4,5].

Once the inspection task and the model have been defined, it is important to treat what will be referred to in later sections as the inspection space. Using set-based methods, which will be described in more detail in later sections, the proposed methods generate a set of valid inspection poses for each facet of the part’s mesh. The set of these poses defines a region in space wherein any camera position or orientation will yield a valid solution to the inspection task, i.e., the given facet will be properly inspected. This will be called the inspection space. The cloud of yellow boxes in Figure 1 shows what this inspection space would look like for a given facet of interest. Intersecting these inspection spaces for each facet in future work would give the final deployment solutions to inspect the entire part; however, this is beyond the scope of this paper.

For the work contained herein, the inspection task and space will be defined for a single “facet of interest”, or foi. This is simply the single facet of the part’s triangular mesh that the camera must inspect, and for which inspection poses will be defined according to inspection constraints. While this paper focuses on methods for a single facet of interest, future work will expand on using this methodology to derive inspection spaces for multi-facet inspection tasks.

The last term that must be treated is the sensor. The sensor, in the broadest terms, is the imaging device used to capture data about the part during the inspection task. In this study, it will be considered as a single camera. Other sensors could be treated in future work using the same methodology, but because this research focuses on visual inspection, only cameras will be considered herein. As such, the terms “camera” and “sensor” will be used interchangeably.

Thus, the overall problem can be defined as solving the complete inspection space for a particular model and inspection task pair according to the constraints that will be imposed by the characteristics of the sensor that will be used to carry out the inspection task.

## 3. Existing Literature

While the problem of camera pose generation for observation of an object has been around since the mid-1980s, the first work which bears any significant resemblance to the problem in its current state began to appear in the mid-1990s [1,7]. These methods were primarily concerned with one of three tasks: object recognition, scene reconstruction, and feature detection [7]. Of these, feature detection and inspection is the area from which the sensor deployment problem would arise.

These early methods also quickly established the importance of several aspects of the problem which need to be considered in order to adequately address the inspection problem, particularly the identification and accurate representation of the sensor and object models [7]. However, the most important by far is proper modelling of the sensor, and proper consideration of the constraints these sensor characteristics impose on the relative positions of the sensor and the object in order to properly satisfy the inspection task, i.e., modelling the constraints on the camera pose for task satisfaction.

### 3.1. Sensor Modelling

**Figure 2:**
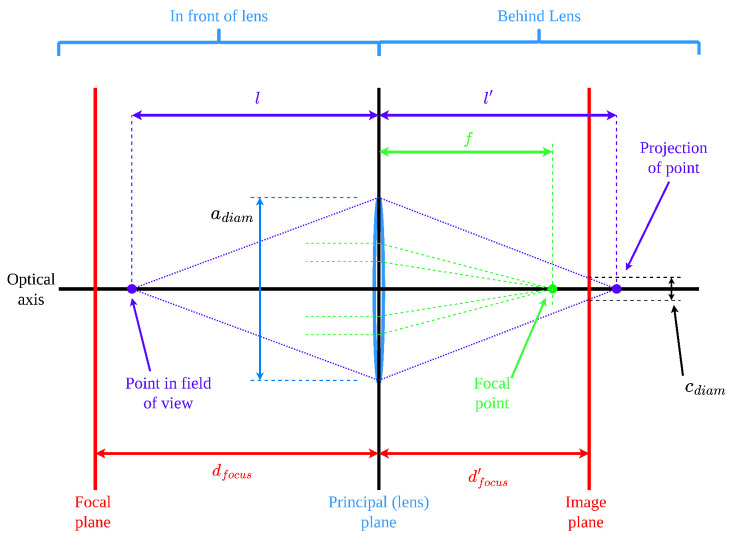
Thin lens model.

Modelling of the sensor is accomplished most simply in the literature through the use of a pinhole camera model [1,4,5,8,9], which is analogous to the very earliest camera obscura in which light simply passes through a very small hole onto the image capture plane. This model is most often chosen for its simplicity, as its most basic form only requires two parameters: the image principal point, *p*, and the focal length, *f*. This model is often considered accurate enough for most applications [8], but for this research, it is insufficient. Thus, this model will be abstracted to the thin lens case, which assumes an infinitesimally thin lens with a given aperture diameter [10]. This requires only one additional parameter, the aperture diameter. This additional parameter allows for the modelling of real effects such as image blur [5]. The thin lens model is illustrated in Figure 2. While other more complex models such as the thick lens model [2,7,10] and the affine sensor model [11] exist, their complexity is beyond the scope of this paper.

### 3.2. Constraint Modelling

From very early work in the field, it was agreed that there are three main criteria which must be met in order to have an image of a part, or a feature thereof, properly satisfying the inspection task requirements [2,3,4,5,7]. They are:

- The part or feature must be visible within the image;
- The part or feature must be in focus;
- The part or feature must be imaged with a suitable resolution.

The visibility constraint implies exactly that; the part feature in question must be visible from the viewpoint of the sensor. This means that it must not be occluded in any way by other part features or external geometries, and generally, that the angle between its normal and the negative of the sensor viewing axis must be less than 90 degrees [3], i.e., backface culling. This also includes a field of view constraint, which is related to a given sensor’s maximum field of view angle. The focus constraint dictates that the part or feature must be within a given distance from the sensor such that the image is sufficiently sharp (this is usually expressed in terms of blur circle radius, which will be discussed in more detail in later sections) [4]. Finally, the resolution criteria is based on the pixel resolution of the imaging sensor. It requires that the projection of the part or feature in the image have a minimal size (in pixels) such that it can be examined with a sufficient level of detail for the inspection task [7,12], and is accounted for by the distance and viewing angle constraints in this work [4].

How researchers attempted to satisfy these constraints has continuously evolved over the years. Some of the earliest methods used minimum radius geodesic domes [1,7,13] constructed around the object in question to ensure the sensor was always at an appropriate distance to satisfy the focus constraint. This dome was also constructed such that the part would always be at its centre, fully within the field of view, and imaged at an acceptable level of resolution. Finally, they used ray tracing from proposed viewpoints on the dome surface in order to ensure visibility [1], although newer methods suggest using sampled point clouds and various statistical filtering techniques to determine the presence of occluding geometry [14]. These methods were also implemented on parametric models of the target part. Additionally, these early methods tended to fall under what is referred to in the literature as “generate and test” strategies, in which sensor configurations are generated via less formal heuristics and gradually improved [7,15].

However, once the use of tesselated part models became the standard, methods began to evolve in order to suit this new representation. The most notable improvement was the shift from heuristic based “generate and test” methods towards more mathematically rigorous “synthesis” methods. These methods, as in [2,3,4,5,16,17,18,19,20,21] attempt to frame the constraints as mathematical functions relating camera position and feature characteristics so as to have a more quantifiable relationship between the two. This includes using triangle normals to help determine camera rotations and visibility [2,3,4,5], as well as spatial triangle coordinates to determine focus, field of view, and resolution. Framing the constraints as algebraic expressions also allows for them to be used as constraints in more computationally modern optimization routines, which is how most modern approaches attempt to solve the problem. For instance, Ref. [2] makes use of recursive solvers, Ref. [3] uses hierarchical genetic algorithms, Refs. [16,17,18] use artificial neural networks and fuzzy inference systems, Ref. [19] makes use of Parisian algorithms, and Refs. [4,5,22] use convex optimization methods such as genetic algorithms and particle swarm optimization. While these methods have all shown promising results, they are all limited by their generation of discrete point solutions with no ability to account for uncertainty in modelling or deployment, which is the gap in the literature which this research addresses.

## 4. Camera Models

Given that the problem is primarily concerned with how well a camera can inspect a particular facet in a triangular mesh, one must first establish the camera model that will be used in the formulation of the problem. This is done to understand how the camera will capture an image and how this will inform the constraints that define whether or not an object is suitably inspected from a given pose.

While a large portion of the literature on optimal sensor placement assumes simple pinhole camera models, these models are overly simplistic, and thus not realistic enough for the deployment of real cameras. As such, the camera model assumed in this research is the thin lens approximation model. This model assumes an aperture with a finite diameter, along with an infinitesimally thin ideal lens [10].

The first basic aspect of the model that must be defined is the optical axis. The optical axis is the presumed axis that passes through the centre of the lens and the image centre [23]. Next, the principal plane of the camera model is defined as the plane normal to the camera axis which intersects it at the lens [10,24]. The final basic aspect of the model is the focal point, which is defined in [10] as the point along the optical axis which has the property of any rays passing through it into the lens will be refracted parallel to the optical axis after passing through the lens. These three elements are illustrated in Figure 2.

The distance between the camera’s principal plane and the focal point is referred to as the focal length, *f*. The focal point is defined as the point behind the lens at which all rays passing through the lens parallel to the optical axis converge [10]. This, coupled with the distance between an object and the lens, *l*, and the distance from the lens to the image plane, $l^{\prime }$, form the basis for the basic equation describing image formation [24], as presented in Equation (1).

(1) $$\frac{1}{l}+\frac{1}{l^{\prime }}=\frac{1}{f}$$

This can be rearranged to represent the distance at which the projection of a given object will converge behind the lens as

(2) $$l^{\prime }=\frac{fl}{l-f}.$$

The next key parameter in the model that is derived from lens characteristics is the $f_{stop}$, which is the ratio of focal length to aperture diameter, $a_{diam}$, expressed as

(3) $$f_{stop}=\frac{f}{a_{diam}}.$$

The aperture diameter is the diameter of the circular opening at the front of the lens assembly which controls the amount of light let through the lens [10]. Expanding on Equation (2), one can determine the relationship between the locations of the focal plane and the image plane (the focal plane is the plane in front of the lens in which an object will be projected perfectly onto the image plane behind the lens [10], see Equation (2) to be

(4) $$d_{focus}^{\prime }=\frac{fd_{focus}}{d_{focus}-f}.$$

The final sensor model concept that will be used in constraint generation is the blur circle, or circle of confusion [5]. This phenomenon is the circular blurring that can be seen in an image around an object when it is not perfectly in focus. The blur circle is the result of the projection of the object in question being either in front of or behind the image plane, which results in it being projected as a circle as opposed to a point. The diameter of the blur circle is expressed as

(5) $$c_{diam}=a_{diam}\frac{l^{\prime }-d_{focus}^{\prime }}{l^{\prime }}.$$

Blur can also be expressed as the blur angle (denoted as $\theta_{blur}$), which is expressed in [10] as

(6) $$\tan\frac{\theta_{blur}}{2}=\frac{c_{diam}/2}{d_{focus}^{\prime }}.$$

By leveraging the small angle identity $\tan\frac{\theta_{blur}}{2}\approx \frac{\theta_{blur}}{2}$ and substituting Equation (6) into Equation (5), along with some rearranging, Ref. [10] showed that $\theta_{blur}$ can then be expressed as

(7) $$\theta_{blur}=a_{diam}\left(\frac{1}{d_{focus}^{\prime }}-\frac{1}{l^{\prime }}\right).$$

These blur quantities are useful, as they will allow the formulation of upper and lower limits on the distance a given object can be from the focal plane while also remaining sufficiently in focus in the final image to allow for adequate inspection.

We must also consider the projection of points in front of the camera onto the camera’s image plane. The image plane is the available surface of the camera’s sensor and is bound in pixel space by the sensor’s height *h* and width *w*. We must also consider the pixel aspect ratio δ, sensor skew *s*, and camera focal length *f*. Altogether, these let us define the camera intrinsic matrix K as

(8) $$K=\left(\begin{array}{ccc} f & s & c_{x}\\ 0 & \delta f & c_{y}\\ 0 & 0 & 1 \end{array}\right).$$

In K, $\left(c_{x},c_{y}\right)$ is the ordered pair defining the image projection centre in pixel space. With an ideal lens, this would mean $\left(c_{x},c_{y}\right)=\left(\frac{w}{2},\frac{h}{2}\right)$, but with real lens aberrations, these values may be slightly different from their ideal values, and these offsets are usually derived via camera calibration algorithms. Additionally, the aspect ratio δ is simply the ratio of pixel height to pixel width, and the sensor skew *s* describes the degree of misalignment of the camera sensor and image plane.

Thus, a point $o_{W}$ in world space

(9) $$o_{W}=\left(\begin{array}{c} x\\ y\\ z \end{array}\right)$$

will be projected into the camera’s pixel space on the image plane as $o_{C}$

(10) $$\left(\begin{array}{c} o_{C}\\ 1 \end{array}\right)=K\left(R|T\right)\left(\begin{array}{c} o_{W}\\ 1 \end{array}\right)$$

where (R|T) is the homogeneous transformation matrix defining the point relative to the world and camera frames.

## 5. Set-Based Methods

This section aims to present some of the fundamental concepts that will frame the novel set-based approach to the optimal inspection problem. It will cover the basics of interval analysis theory, the basics of set-based pose representations, and some simple examples of interval analysis being used to solve constraint problems.

### 5.1. Interval Analysis Methods

This research takes a novel approach to solve the optimal sensor locations by applying interval analysis methods. Originally developed in the 1960s to address the inability of computers to exactly represent most numbers [25], these methods find extensions to standard point number mathematical operations using interval values instead of discrete exact values. By treating numbers as intervals, one can account for rounding and measurement errors in calculations and produce ranges of solutions that are guaranteed to contain the true solution to the given problem. Intervals in this system are represented as

(11) $$\left[x\right]=\left[\underline{x},\bar{x}\right]=\left\{x\in R|\underline{x}\leq x\leq \bar{x}\right\}$$

where $\underline{x}$ and $\bar{x}$ are the lower and upper bounds of the interval, respectively.

Other useful components of intervals are their midpoint,

(12) $$\mathrm{mid}\left(\left[x\right]\right)=\left(\bar{x}+\underline{x}\right)/2$$

and their width,

(13) $$\mathrm{wid}\left(\left[x\right]\right)=\bar{x}-\underline{x}.$$

Other fundamental properties of intervals are discussed in detail in [25].

It is also useful to characterize the interactions between multiple intervals. The two key operations for doing so are the intersection of two intervals,

(14) $$\left[x\right]\cap \left[y\right]=[\max\left(\underline{x},\;\underline{y}\right),\;\min\left(\bar{x},\;\bar{y}\right)]$$

and the hull, or interval union, of two intervals

(15) $$\left[x\right]\cup \left[y\right]=[\min\left(\underline{x},\;\underline{y}\right),\;\max\left(\bar{x},\;\bar{y}\right)].$$

A detailed discussion of interval extensions to standard arithmetic operations can also be found in [25]. Interval extensions of functions typically require that the function be monotone, although there are interval extensions of non-monotonic functions [25].

This leads to the fundamental theory of interval analysis, which states that “the interval extension of a monotonic function f([x]) yields the inclusion function [f], such that f([x]) is contained inside of [f] [26],”

(16) f([x])={f(x)∣x∈[x]}⊆[f].

These interval methods can also be extended in order to describe vectors and matrices of intervals. An interval vector represents an ordered *n*-tuple of intervals [25]

(17) $$\left[x\right]=\left[\left[x_{1}\right],\left[x_{2}\right],\ldots ,\left[x_{n}\right]\right]=\left[\left[\underline{x_{1}},\;\bar{x_{1}}\right],\;\left[\underline{x_{2}},\;\bar{x_{2}}\right],\ldots ,\;\left[\underline{x_{n}},\;\bar{x_{n}}\right]\right].$$

By extension, an interval matrix is represented as

(18) $$\left[A\right]=\left(\begin{array}{cc} \left[\underline{a_{11}},\;\bar{a_{11}}\right] & \left[\underline{a_{12}},\;\bar{a_{12}}\right]\\ {}[\underline{a_{21}},\;\bar{a_{21}}] & \left[\underline{a_{22}},\;\bar{a_{22}}\right] \end{array}\right).$$

These interval methods are applied to the inspection constraints in order to allow for a continuous evaluation of the space around a given facet in order to certifiably synthesize its entire valid inspection space.

### 5.2. Interval Analysis for Constraint Satisfaction

Interval analysis methods for constraint satisfaction in this research will work based on two principal method classes: simplification, and bisection [25]. Given a system of constraints C([u]), where [u] represents the constraint variables, the two methods are applied consecutively through an iterative branch and bound process. For other in-depth analyses of applications of interval methods for the solving of constraint satisfaction problems, see [25,26,27,28,29,30]. The interval-based constraint satisfaction solver algorithm used here is shown in Algorithm 1.
**Algorithm 1** Interval-based constraint satisfaction solver algorithm1:Initialize system variables [**u**]2:Initialize constraints C([u])3:Initialize empty list of unclassified boxes $L_{u}$4:Initialize empty list of valid boxes $L_{v}$5:Initialize search space by contracting [u] according to C([u])6:Add contracted [u] to end of list $L_{u}$7:**if** $L_{u}=Ø$ **then**8:    Terminate solver and return $L_{v}$9:**else**10:    Pop ${\left[u\right]}_{i}$ from $L_{u}$ and evaluate C(${\left[u\right]}_{i}$)11:    **if** Constraints are fully satisfied **then**12:        Classify ${\left[u\right]}_{i}$ as VALID13:        Add ${\left[u\right]}_{i}$ to back of $L_{v}$14:        **go to** 615:    **else if** Constraints are partially satisfied **then**16:        **if** Widest interval in ${\left[u\right]}_{i}$ is narrower than ϵ **then**17:           Classify ${\left[u\right]}_{i}$ as BOUNDARY18:           Add ${\left[u\right]}_{i}$ to back of $L_{v}$19:           **go to** 620:        **else**21:           Bisect ${\left[u\right]}_{i}$ ${\left[u\right]}_{i_{1}}$ and ${\left[u\right]}_{i_{2}}$22:           Add ${\left[u\right]}_{i_{1}}$ and ${\left[u\right]}_{i_{2}}$ to end of $L_{u}$23:           **go to** 624:        **end if**25:    **else**26:        **go to** 627:    **end if**28:**end if**

#### 5.2.1. Simplification Methods

Simplification methods are heuristic methods whose goal is to reduce any excess width of [u] in C([u]) [25], such as HC4, ACID, 2B and 3B filtering, and Newton methods [25,26,27,28]. This work uses HC4 and ACID methods in order to simplify initial variable search spaces (represented by interval vectors) according to constraints as much as possible prior to the application of bisection methods to further refine the solutions.

These methods work by iteratively applying interval arithmetic to the constraint functions in order to narrow the domains of the variables as much as possible. For instance, HC4 works by applying consecutive iterations of forward arithmetic and backward arithmetic [25] to a tree representation of a system to successively narrow the domain of its variables [28]. For example, in the equation ${\left(\left[x\right]-\left[y\right]\right)}^{2}-\left[z\right]=0$, with [x]∈[0,10], [y]∈[0,4], and [z]∈[9,16]), the forward step yields a result of [−16,91] at the root. Setting the root to [0,0] for the backwards step and refining for [x], we find [x] simplified to [x]∈[0,8]. Application of these steps continues over each variable until a given stopping criteria (usually variable width ϵ) is met [28].

#### 5.2.2. Bisection Methods

Bisection methods split the interval [u] along the dimension *i* into $\left[u_{1}\right]$ and $\left[u_{2}\right]$, as long as the width of [u] is greater than a given threshold ϵ [25]. The union of these sub-intervals is equal to the original interval and, as such, they still represent a continuous evaluation of the solution space. The bisection strategy used herein is known as largest-first [27], in which an interval vector [u] is bisected at the midpoint of its widest component interval, and all other components of the interval vector remain unchained in the resultant child interval vectors. These bisections continue until the widths of all components of [u] are below a given threshold, or [u] is found to either fully satisfy constraints or not represent a valid solution. Bisected intervals are added to the list $L_{u}$ [27].

## 6. Set-Based Extensions of Pose and Inspection Constraints

A camera position and orientation are described using x,y,z coordinates and ZXZ Euler angles (φ,γ,β), respectively. Together the position and orientation define the camera pose.

The goal of the set-based constraint formulation is to derive the sets of camera poses such that all points on a given facet are visible from the camera, and the camera specification and other inspection constraints are satisfied. That is, the set of poses P that ensure the facet can be properly inspected is given by

(19) $$\begin{array}{c} P=\{\left(x,y,z,\varphi ,\gamma ,\beta \right)|C_{k}\left(x,y,z,\varphi ,\gamma ,\beta \right)\;\mathrm{is}\;\mathrm{valid},\\ x,y,z\in R,\varphi ,\gamma ,\beta \in [-2\pi ,2\pi ],k=1..n\} \end{array}$$

where $C_{k}\left(x,y,z,\varphi ,\gamma ,\beta \right)$ is one of *n* inspection constraints.

Let a camera pose interval be given by $\left[p\right]=(\left[x\right],\left[y\right],\left[z\right],\left[\varphi \right],\left[\gamma \right],{\left[\beta \right]}_{i})$ for a given facet. The pose solution guarantees that the entire facet satisfies the considered constraints ∀p∈[p],[p]∈P.

### 6.1. Set-Based Representation of Poses

The two elements of pose which must be addressed are position and orientation. The position of a point in the world frame $o_{W}$, and the set-based extension of this vector, are based on imposing upper and lower uncertainty limits [31] on each element of the vector such that each is transformed to be

(20) $$\begin{array}{cc} \left[x\right] & =[\underline{x},\;\bar{x}] \end{array}$$

(21) $$\begin{array}{cc} \left[y\right] & =[\underline{y},\;\bar{y}] \end{array}$$

(22) $$\begin{array}{cc} \;\left[z\right] & =[\underline{z},\;\bar{z}]. \end{array}$$

Thus, $o_{W}$ becomes $\left[o_{W}\right]$, where

(23) $$\left[o_{W}\right]=\left(\begin{array}{c} \left[x\right]\\ \left[y\right]\\ \left[z\right] \end{array}\right)$$

where $\left[o_{W}\right]$ now represents all of the points contained inside a 3D box in position space, as opposed to the discrete point represented by $o_{W}$.

This research will express orientation as a vector of ZXZ Euler angles (φ, γ, β) [32] as

(24) $$e=\left(\begin{array}{c} \varphi \\ \gamma \\ \beta \end{array}\right)$$

**Figure 3:**
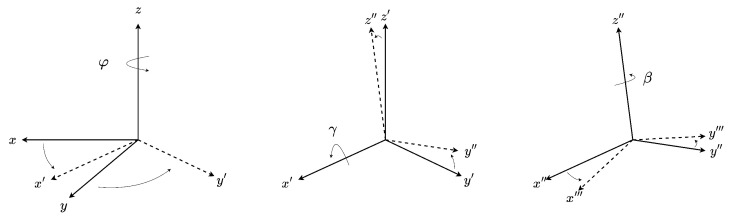
Orientation rotation sequence.

This representation is chosen due to its ease of bisection, and its compatibility with orientation constraints. These rotations are applied successively such that the rotation matrix of cumulative rotation operation can be expressed as

(25) $$R_{e}=R_{z}\left(\varphi \right)R_{x^{\prime }}\left(\gamma \right)R_{z^{\prime \prime }}\left(\beta \right)$$

and is demonstrated in Figure 3.

This ZXZ representation can easily be extended to a set-based context using intervals much like the position was in Equation (23) such that e becomes [e],

(26) $$\left[e\right]=\left(\begin{array}{c} \left[\varphi \right]\\ \left[\gamma \right]\\ \left[\beta \right] \end{array}\right)=\left(\begin{array}{c} \left[\underline{\varphi },\;\bar{\varphi }\right]\\ {}[\underline{\gamma },\;\bar{\gamma }]\\ \underline{\left[\beta \right]},\;\bar{\beta ]}] \end{array}\right).$$

Thus, [e] now represents a 3D box in rotation space as opposed to the single finite point represented by e. Together, $\left[o_{W}\right]$ and [e] represent a 6D box in pose space that describes a continuous set of poses.

The position and orientation intervals are solved as two separate constraint systems in this methodology, along with a third system for determining if any derived poses result in the camera’s view of the foi being occluded by any objects within the scene. Since the occlusion test is orientation-independent, it is grouped with position constraints but is solved by a separate system of constraints within them.

### 6.2. Position Constraints

The constraints that must be solved for a given pose box [p] in the main position constraint system $C_{p}$ for a facet are:

1. Does [p] intersect the facet?
2. Is [p] an appropriate distance from the facet?
3. Is [p] in front of the facet?
4. Does [p] inspect the facet from a suitable angle?

First, to test if [p] intersects the facet, we consider the set of all points on the surface of the facet as the region bounded by the set of plane inequality constraints $C_{f}$. $C_{f}$ is defined by the 3D plane that contains the facet, and the three planes perpendicular to it, which each contain one of the edges of the facet. We can then say

(27) $$C_{f}\cap \left[p\right]\neq Ø,\;\left[p\right]\;\mathrm{is}\;\mathrm{not}\;\mathrm{valid}$$

(28) $$C_{f}\cap \left[p\right]=Ø,\;\left[p\right]\;\mathrm{satisfies}\;\mathrm{constraint}\;1,\;\mathrm{continue}.$$

To solve for the valid set of [x],[y],[z] positions in [p] for the facet bounded by $C_{f}$, the following distance constraint is required:

(29) $$C_{distance}={\left(\begin{array}{c} \left[x\right]\\ \left[y\right]\\ \left[z\right] \end{array}\right)}^{2}-{\left[c\right]}_{f}^{2}\subseteq \left[d_{min}^{2},d_{max}^{2}\right]$$

where $d_{min}$ and $d_{max}$ are constants defining the minimum and maximum depth of field values for the image of the facet to be suitably in focus for a given inspection camera, and ${\left[c\right]}_{f}$ is the interval vector containing the valid solutions to $C_{f}$. The $d_{min}$ and $d_{max}$ parameters are derived according to lens intrinsic parameters. They determine how far away from the facet a camera can be while still satisfying inspection requirements.

The constraint for testing whether a box [p] is in front of a facet is called the backface constraint, and is evaluated by creating a half-space constraint defined by the plane containing the facet. Using the facet’s normal, $n={\left[n_{x},n_{y},n_{z}\right]}^{T}$, and one vertex, $v={\left[v_{x},v_{y},v_{z}\right]}^{T}$, we can define the constraint as

(30) $$\left[p\right]=\left\{\begin{array}{cc} \mathrm{in}\;\mathrm{front}: & \mathrm{if}\;([p]-v)\cdot n>0\\ \mathrm{intersecting}: & \mathrm{if}\;0\in ([p]-v)\cdot n\\ \mathrm{behind}: & \mathrm{if}\;([p]-v)\cdot n<0. \end{array}\right.$$

Finally, to determine if the viewing angle is sufficiently large for the foi to be inspectable, we define the constant $\theta_{v}$ as the minimum viewing angle, and the facet’s geometric centre as $f_{j_{c}}$. The constraint is then

(31) $$\arccos\left(\left({\left[p\right]}_{i}-f_{j_{c}}\right)\right)\cdot n\leq \left(\frac{\pi }{2}-\theta_{v}\right).$$

Once box [p] has been shown to satisfy these position constraints, it must be tested to ensure that no position in it represents one whose view of $f_{j}$ would be occluded by any other geometry within the scene.

### 6.3. Occlusion Testing

#### 6.3.1. Underlying Methodology

Once the three-dimensional position box [p] has been solved for the facet of interest, it must then be checked to verify that it does not contain positions for which the view of the facet is occluded by any other geometries (either internal, i.e., other facets belonging to the part, or external, i.e., by other objects/geometries present in the inspection space). Note that only the [x],[y],[z] components of [p] are considered in this test, as it is primarily concerned with determining if there is any straight continuous path between any point on the facet and any point in the position box which intersects other geometries. The occlusion testing process will be demonstrated herein in 2D, but the techniques in question are easily extrapolated into 3D.

**Figure 4:**
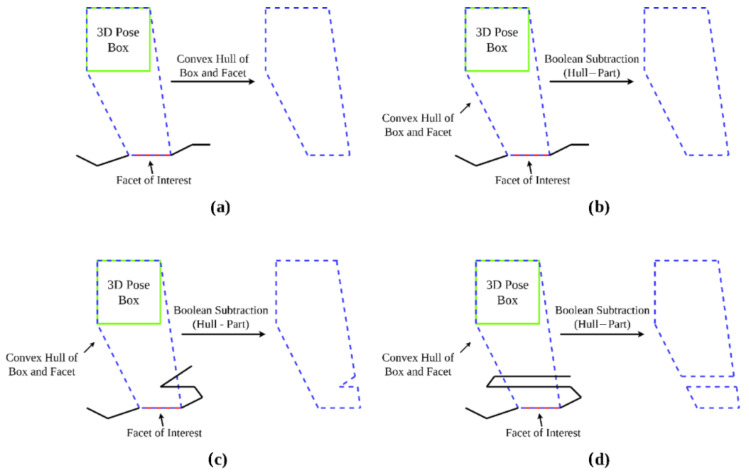
(**a**) Pose box and facet of interest; (**b**) differencing result for no occlusion; (**c**) differencing result for partial occlusion; (**d**) differencing result for full occlusion.

The first step in the occlusion testing algorithm is to build the convex hull containing both [p] and the facet of interest (this will be called the camera mesh) as shown in Figure 4a. This convex hull is constructed, and further operations are conducted, using exact predicates and geometric operations with CGAL such that it can be certified that any results are an exact computation of any further mesh boolean operations.

The scene mesh is then subtracted from this camera mesh, and the visibility of the facet of interest from [p] can then be quantified based on the result of this boolean difference operation.

The first test case will be for a set of camera positions for which the facet will be fully visible. Figure 4b shows the camera mesh and the part mesh before the differencing operation, as well as the resultant mesh. As the box represents positions from which the facet is fully visible, the camera mesh remains unchanged, as one would expect.

Next, a box in which some, but not all, positions within the set have another geometry occluding the facet is presented. Figure 4c shows the camera mesh and the part mesh, along with the resultant mesh, before and after the differencing operation.

From these, it is plain to see the effect that the differencing operation has had on the camera mesh, as the section of the part mesh that intersected the camera mesh has been subtracted from the initial camera mesh. The resultant void causes the differenced mesh to have more facets than the original camera mesh, which tells the algorithm that at least some degree of occlusion is present. One can say that the occlusion is partial rather than full, because while the differenced mesh is discontinuous, the vertices corresponding to the camera position box and those corresponding to the facet of interest are still part of the same continuous sub-mesh, and there are still edges remaining which connect at least one box vertex to at least one facet vertex.

Finally, a box for whom all inner positions represent an occluded view is presented. Figure 4d shows the camera mesh, the part mesh, and the resultant mesh.

Because the mesh subtracting has resulted in two separate meshes, which separately contain the original box vertices and the original facet vertices, the algorithm will classify this result as a case in which the particular facet of interest is fully occluded from any viewpoint within the original pose box. While this is the most common case for a full occlusion, there is a second case to consider: the case in which the subtraction still results in one continuous mesh, but one which does not contain any of the original facet vertices. This would be the case for a box that was behind the plane of the facet of interest, which, while possible, would be filtered out as a possible valid set of poses by the previously described backface culling condition.

#### 6.3.2. Facet Occlusion Classification

The algorithm for classifying the visibility of the facet of interest from [p] using the above methods is described in Algorithm 2.

**Figure 5:**
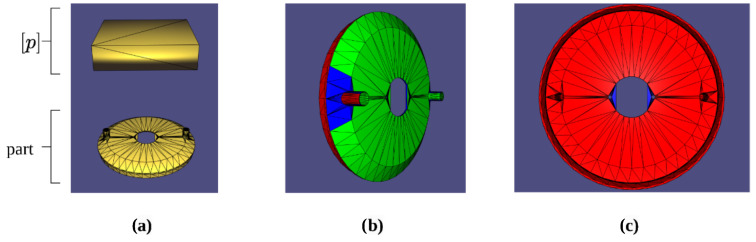
(**a**) Part and test box; (**b**) occlusion classifications with detail around one of the top surface protrusions; (**c**) occlusion classifications on the bottom surface of part.

The results of this algorithm for classifying the visibility of facets on the sample part for the box described in Equation (32) as

(32) $$\left[p\right]=\left(\begin{array}{c} {}[-0.1,\;0.1]m\\ {}[-0.1,\;0.1]m\\ {}[0.25,\;0.3]m \end{array}\right)$$

are presented in Figure 5b,c, with the box and part in Figure 5a. In these figures, green represents a fully visible facet, blue represents a partially visible facet, and red represents a fully occluded facet.
**Algorithm 2** Algorithm for determining if facet is occluded1:Generate convex hull containing all facet and [p] vertices (camera mesh)2:Generate convex hull containing just [p] vertices (box mesh)3:Subtract part mesh from camera mesh4:Compare differenced camera mesh to initial camera mesh5:**if** Identical **then**6:    facet is fully visible7:**else**8:    **go to** 109:**end if**10:Check if camera mesh contains all original facet/box vertices11:**if** Differenced camera mesh contains no original box vertices, subtract part mesh from initial box mesh **then**12:    **if** Result is empty **then**13:        $f_{j}$ is not visible14:    **else**15:        **go to** 2016:    **end if**17:**else if** Differenced camera mesh does not contain all facet vertices **then**18:    facet is not visible19:**end if**20:Check if facet vertices and remaining box vertices (or vertices of differenced box mesh if no original box vertices are present) are connected in differenced camera mesh21:**if** yes **then**22:    facet is partially visible23:**else**24:    facet is not visible25:**end if**

### 6.4. Orientation Constraints

The orientation of the camera from any given position such that the facet is within its field of view is primarily determined by the camera’s field of view (FOV) half angles. Let the FOV half angles be $\alpha_{v}$ and $\alpha_{h}$ for the vertical and horizontal axes, respectively. The camera axis c is given by

(33) $$\left[c\right]=\left[R\left(\left[\varphi \right],\left[\gamma \right],\left[\beta \right]\right)\right]\hat{z}$$

where

(34) $$\hat{z}=\left(\begin{array}{c} 0\\ 0\\ 1 \end{array}\right).$$

A FOV constraint $C_{FOV}\left(x,y,z,\varphi ,\gamma ,\beta \right)$ is formulated to ensure that the entire facet is visible from the camera pose.

Before constraint systems can be generated and solved to define complete orientation intervals, a few transformations must be made in order to simplify the problem and allow for a more efficient solving. The formulation of the orientation limits for a given box corresponding to a given facet begins by considering the facet vertices and the 3D box defining a set of solutions for camera position. The facet vertices are presented in a matrix of discrete values, $F_{v}$, as

(35) $$F_{v}=\left(\begin{array}{ccc} v_{1_{x}} & v_{2_{x}} & v_{3_{x}}\\ v_{1_{y}} & v_{2_{y}} & v_{3_{y}}\\ v_{1_{z}} & v_{2_{z}} & v_{3_{z}} \end{array}\right).$$

The solver also requires the camera position box, [p], and facet bounding box ${\left[o\right]}_{f}$.

The difference equations in Equations (36) and (37) are then applied in order to transform the facet and vertex boxes such that instead of attempting to determine the relationship between two boxes, we can examine the positions of the boxes relative to a common discrete origin. The difference of the facet and camera boxes is referred to as ${\left[d\right]}_{f}$ and that of the vertices and the camera is ${\left[D\right]}_{v}$.

(36) $${\left[d\right]}_{f}={\left[o\right]}_{f}-\left[p\right]$$

(37) $${\left[D\right]}_{v}=F_{v}-\left(\left[p\right],\left[p\right],\left[p\right]\right)$$

By then taking the midpoint of ${\left[d\right]}_{f}$, one can solve for a nominal camera vector $c_{nom}$ originating at the centre of ${\left[p\right]}_{i}$ and passing through the facet geometric centre. If we consider this as the *z*-axis of a nominal camera frame, we can use simple transformations to solve for the first two components of a *ZXZ* Euler angle rotation sequence, which results in a nominal camera frame oriented towards the facet from [p]. We call these values $\varphi_{nom}$ and $\gamma_{nom}$, and we set $\beta_{nom}=0$.

Finally, we apply the *ZXZ* Euler rotation sequence defined by $R(\varphi_{nom},\gamma_{nom},\beta_{nom})$ to the columns of ${\left[D\right]}_{v}$,

(38) $${\left[D\right]}_{v_{1:3,i}}^{\prime }=R\left(\varphi_{nom},\gamma_{nom},\beta_{nom}\right){\left[D\right]}_{v_{1:3,i}},\;i=1\ldots 3$$

in order to place the vertices of the differenced box into camera space. We call this transformed matrix ${\left[D\right]}_{v}^{\prime }$. From here, we can define two constraint systems which will solve the orientation components. It should be noted that the first constraint system is solving for the allowable offsets of [φ] and [γ] (${\left[\varphi \right]}_{offset}$ and ${\left[\gamma \right]}_{offset}$) about their nominal values ($\varphi_{nom}$, $\gamma_{nom}$), and the final [φ] and [γ] intervals will be the sum of the offset intervals and the nominal values. However, the second constraint system solves for [β] directly.

#### 6.4.1. Allowable ${\left[\varphi \right]}_{offset}$ and ${\left[\gamma \right]}_{offset}$ Intervals

If we look at each rotation component separately and consider the corresponding FOV half angles, we can create a set of four hyperplanes passing through the origin which bound the points in ${\left[D\right]}_{v}^{\prime }$ from Equation (38). These four hyperplanes are split into two pairs, with one pair parallel to the camera frame’s *XZ*-plane, and the other parallel to the camera frame’s *YZ*-plane. By rotating each in opposite directions about the camera’s *X*- or *Y*-axis, respectively, we create an artificial “frustum” which bounds the vertices of ${\left[D\right]}_{v}^{\prime }$. By examining the angles between each hyperplane and its original plane in the camera frame, we can determine the allowable offsets about $\varphi_{nom}$ and $\gamma_{nom}$. The variables involved in this constraint system are the four hyperplane angles,

1. $\left[\varphi_{left}\right]\in \left[0,\pi \right]$
2. $\left[\varphi_{right}\right]\in \left[-\pi ,0\right]$
3. $\left[\gamma_{up}\right]\in \left[-\pi ,0\right]$
4. $\left[\gamma_{down}\right]\in \left[0,pi\right]$

along with the constants

1.
$\alpha_{h}$

2.
$\alpha_{v}$

3. ${\left[D\right]}_{v}$.

This, in turn, leads to the constraints

(39) $${\left[D\right]}_{v_{i_{x}}}\cos\left[\varphi_{left}\right]+{\left[D\right]}_{v_{i_{z}}}\sin\left[\varphi_{left}\right]\leq 0$$

(40) $${\left[D\right]}_{v_{i_{x}}}\cos\left[\varphi_{right}\right]+{\left[D\right]}_{v_{i_{z}}}\sin\left[\varphi_{right}\right]\geq 0$$

(41) $${\left[D\right]}_{v_{i_{y}}}\cos\left[\gamma_{up}\right]+{\left[D\right]}_{v_{i_{z}}}\sin\left[\gamma_{up}\right]\leq 0$$

(42) $${\left[D\right]}_{v_{i_{y}}}\cos\left[\gamma_{down}\right]+{\left[D\right]}_{v_{i_{z}}}\sin\left[\gamma_{down}\right]\geq 0$$

(43) $$|\left[\varphi_{right}\right]-\left[\varphi_{left}\right]|\leq \alpha_{h}$$

(44) $$|\left[\gamma_{up}\right]-\left[\gamma_{down}\right]|\leq \alpha_{v}.$$

By then using HC4 and ACID contractors to contract the interval variables, we can solve for their domains,

(45) $${\left[\varphi \right]}_{offset}=\left[-\alpha_{h}+\underline{\varphi_{left}},\alpha_{h}+\bar{\varphi_{right}}\right]$$

and

(46) $${\left[\gamma \right]}_{offset}=\left[-\alpha_{v}+\underline{\gamma_{down}},\alpha_{v}+\bar{\gamma_{up}}\right].$$

Having solved for the domains, we can then define the full allowable φ and γ intervals for the given pose box as

(47) $$\left[\varphi \right]=\varphi_{nom}+{\left[\varphi \right]}_{offset}$$

(48) $$\left[\gamma \right]=\gamma_{nom}+{\left[\gamma \right]}_{offset}.$$

#### 6.4.2. Allowable β Interval

Once allowable [φ] and [γ] intervals have been calculated for a given pose box, it is possible to identify the allowable [β] angle interval for a given set of camera poses. The process starts with ${\left[D\right]}_{v}^{\prime }$. We can then solve for the projection of each of the vertices (the columns of ${\left[D\right]}_{v}^{\prime }$) onto the camera’s image plane. For each column ${\left[d\right]}_{v_{i}}^{\prime }$, i=1…3 in ${\left[D\right]}_{v}^{\prime }$, we can use the matrices K and [R([φ],[γ],[β])] to get the projection ${\left[d\right]}_{p_{i}}$,

(49) $${\left[d\right]}_{p_{i}}=K\left[R\left(\left[\varphi \right],\left[\gamma \right],\left[\beta \right]\right)\right]{\left[d\right]}_{v_{i}}^{\prime },\;i=1\ldots 3.$$

Because K and ${\left[d\right]}_{v_{i}}^{\prime }$ are constant matrices, and we have already solved for [φ] and [γ], we can then define the constraints

(50) $$K\left[R\left(\left[\varphi \right],\left[\gamma \right],\left[\beta \right]\right)\right]{\left[d\right]}_{v_{i}}^{\prime }>{\left(0\right)}_{3\times 1}$$

(51) $${\left(K\left[R\left(\left[\varphi \right],\left[\gamma \right],\left[\beta \right]\right)\right]{\left[d\right]}_{v_{i}}^{\prime }\right)}_{1:2}<\left(\begin{array}{c} w\\ h \end{array}\right).$$

then subsequently apply a combination of HC4 and ACID contractors to contract the domain of [β], such that the constraints are satisfied.

## 7. Overall Pose Solver Algorithm

Using all of the concepts established herein, it is now possible to formulate an overall pose solver algorithm for a single facet in an arbitrary 3D mesh representing a real part geometry. First, the initial constraints are formulated for determining whether or not a given set of poses represents a valid solution to the inspection pose problem. This includes distance constraints, orientation/field of view constraints, and visibility and occlusion constraints. Next, using interval-based system contraction methods, the search space around the part is contracted to find the initial position search space, which is the axis-aligned bounding box that most tightly bounds all possible solutions. From there, the position constraints are evaluated over this box, and it is iteratively subdivided and analyzed in order to determine the full set of valid, boundary, and invalid position boxes within the initial box. Next, the boxes are tested for any possible occlusion of a camera’s view of the facet from that box by any other geometries present in the scene. Finally, these position boxes have their respective orientation intervals evaluated as per the method in Section 6.4, and are subsequently refined/subdivided should they be too wide for the camera’s field of view. Once all pose intervals (position and orientation) for each box have been solved and all boxes have been classified, the full tree structure containing all boxes is returned as the final synthesized solution set of all possible valid inspection poses for the facet of interest. This process is detailed in Algorithm 3.
**Algorithm 3** Overall algorithm for determining a complete set of valid inspection poses for single facet1:Initialize empty list of unclassified position boxes $L_{u}$2:Initialize empty list of valid position boxes $L_{v}$3:Initialize empty list of solution 6D pose boxes $L_{s}$4:Initialize search space by contracting position variables to get the initial box [u]5:Add box to end of list $L_{u}$6:**if** $L_{u}\neq Ø$ **then**7:    Evaluate position and occlusion constraints8:    **if** Constraints are fully satisfied **then**9:          Classify box ${\left[k\right]}_{i}$ as VALID10:        Add ${\left[k\right]}_{i}$ to back of $L_{v}$11:        **go to** 612:    **else if** Constraints are partially satisfied **then**13:        **if** Widest interval in ${\left[u\right]}_{i}$ is narrower than minimum resolution **then**14:           Classify box ${\left[u\right]}_{i}$ as BOUNDARY15:           Add box ${\left[k\right]}_{i}$ to back of $L_{v}$16:           **go to** 617:        **else**18:           Bisect ${\left[u\right]}_{i}$ into ${\left[u\right]}_{i_{1}}$ and ${\left[u\right]}_{i_{2}}$19:           Add ${\left[u\right]}_{i_{1}}$ and ${\left[u\right]}_{i_{2}}$ back onto back of $L_{u}$20:           **go to** 621:        **end if**22:    **else**23:        Remove ${\left[u\right]}_{i}$ from $L_{u}$24:        **go to** 625:    **end if**26:**else**27:    Terminate position solver and return $L_{v}$28:    **while** $L_{v}\neq Ø$ **do**29:        Pop ${\left[k\right]}_{i}$ from $L_{v}$ and solve orientation intervals according to Section 6.430:        **if** $\left({\left[\varphi \right]}_{k_{i}}>\alpha_{h}\right)|\left({\left[\gamma \right]}_{k_{i}}>\alpha_{v}\right)$ **then**31:           Bisect ${\left[k\right]}_{i}$ ${\left[k\right]}_{i_{1}}$ and ${\left[k\right]}_{i_{2}}$32:           Add ${\left[k\right]}_{i_{1}}$ and ${\left[k\right]}_{i_{2}}$ back onto back of $L_{v}$33:        **else**34:           Add completely resolved ${\left[k\right]}_{i}$ to end of $L_{s}$35:        **end if**36:    **end while**37:    Return $L_{s}$ as a complete set of camera deployment poses for facet of interest38:**end if**

## 8. Single Facet Case Study

**Figure 6:**
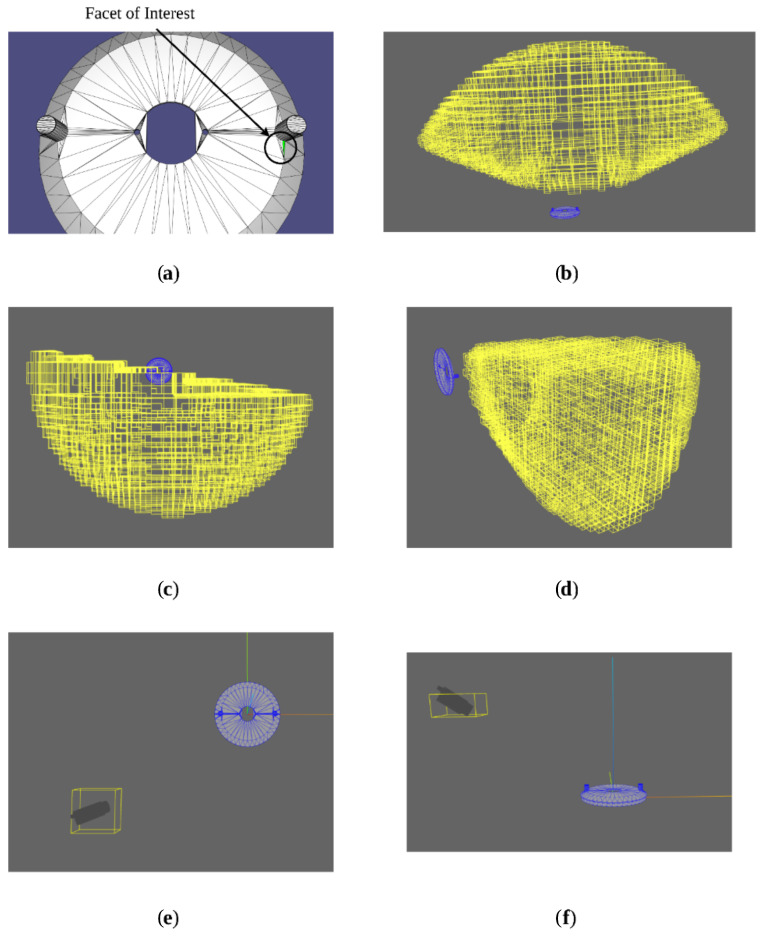
(**a**) Sample facet for pose solver test; (**b**–**d**) various viewpoints of the full solution set; (**e**,**f**) various views of a sample pose box showing nominal camera orientation.

Combining all previous elements, we can now solve for a given facet’s entire set of valid poses. This was tested for the facet highlighted as the facet of interest in Figure 6, as it has obvious occlusions due to the protrusion on the top surface of the part, and as such is a good representative sample for the effectiveness of the visibility test in removing invalid boxes.

The valid position boxes for this facet are presented from various positions in Figure 6b–f show a sample selected pose box with nominal camera orientation so as to effectively demonstrate the full 3D solution space.

The simulation was carried out on a laptop computer with an Intel^®^ Core^TM^ i5-6440HQ CPU @ 2.60 GHz × 4 processor (Intel Corporation, Santa Clara, CA, USA) and 8 GB of RAM. The sample part has a total of 1145 vertices and 2298 faces, with a single face selected as the facet of interest. The test was conducted with a position bisection stopping threshold of 0.1 m, and an orientation bisection stopping threshold of 0.38 rad.

## 9. Discussion of Results and Future Work

The end result of this simulation produced a total of 1614 6D pose boxes. On average, this test took approximately 53 s. It is clearly visible in Figure 6b that the pose solutions obey the maximum distance and viewing angle constraints as expected, while Figure 6d shows the underside of the solution set obeying minimum distance requirements as well. Furthermore, Figure 6c clearly shows the solution set obeying the occlusion constraint, as no boxes are present behind the planes containing the adjacent facets of the protrusion on the top surface which would occlude the view of the facet of interest. Figure 6e,f also shows that the camera is correctly oriented to have the field in its field of view.

It is clear from these results that the algorithm was able to effectively synthesize the complete continuous set of valid camera poses (for a given resolution) for the example facet on the part given the constraints defined in previous sections.

However, the 53-s computational time for a single facet’s complete set of pose solutions does imply that the computational complexity of the algorithm may increase dramatically with the inclusion of more facets and the use of more complex parts. As such, computational optimizations will be a significant focus for future work, but they are beyond the scope of this paper.

## 10. Conclusions

The methodology presented herein establishes the fundamental concepts for synthesizing complete inspection spaces for a given part geometry. This is undertaken by first establishing the methods for synthesizing the continuous inspection space for a single facet from a part’s triangular mesh representation. The set-based constraints which were formulated were able to effectively solve for the complete valid inspection space for the example facet in question, and are a promising preliminary result towards the solving of inspection spaces for a part’s entire geometry. Future work will focus on solving the intersections of individual facet inspection spaces for complete solution synthesis and further refinement of constraints to account for real camera intrinsic parameters, which are considered idealized in this scenario, as well as emphasizing computational efficiency. To the best of the authors’ knowledge, the derived methodology is new and can be readily adopted and scalable in various manufacturing facilities and applications.

## Data Availability

No new data were created or analyzed in this study. Data sharing is not applicable to this article.

## Nomenclature

The following abbreviations are used in this manuscript:

*x*	Scalar
x	Vector
$\hat{x}$	Unit vector
X	Matrix
[x]	Interval variable
[x]	Interval vector
[X]	Interval matrix
${\left\{o\right\}}_{X}$	Coordinate frame
F	Constraint
P	Set
L	List
